# Principles of Network Architecture Emerging from Comparisons of the Cerebral Cortex in Large and Small Brains

**DOI:** 10.1371/journal.pbio.1002556

**Published:** 2016-09-15

**Authors:** Barbara L. Finlay

**Affiliations:** Department of Psychology, Cornell University, Ithaca, New York, United States of America

## Abstract

The cerebral cortex retains its fundamental organization, layering, and input–output relations as it scales in volume over many orders of magnitude in mammals. How is its network architecture affected by size scaling? By comparing network organization of the mouse and rhesus macaque cortical connectome derived from complete neuroanatomical tracing studies, a recent study in *PLOS Biology* shows that an exponential distance rule emerges that reveals the falloff in connection probability with distance in the two brains that in turn determines common organizational features.

## Abstract 2

In both mouse and monkey cortex, the connection probability of cortical regions falls off exponentially with absolute distance; conservation of cortical network organization during mammalian brain evolution could arise naturally from this rule. Read the related Research Article.

How do you scale up a computing device? The mammalian brain has been presented a massive challenge: to retain its basic divisions and connectivity, despite brain volumes ranging from less than a gram in the smallest shrew to over 8,000 grams in the largest whale. In the cerebral cortex alone (isocortex or cortex), the problem is amplified, as the isocortex scales in volume disproportionately with respect to the rest of the brain, especially in primates [1]. Understanding how a computing device might scale would obviously be easier if its computational function were known. The computations the cortex performs are not definitively known, however, though there is no shortage of theories on this subject. Because of the bootstrapping nature of research on evolution, a description of how the brain adapts its connectivity to massive increases in numbers of neurons, however, may shed light on its essential computational role. The organizational features the cortex has defended over several hundred million years and massive size differences are important clues to its essential computations.

The isocortex in mammals is a layered sheet of neurons that, according to one view, offers considerable uniformity. Within any particular cortex, and across species, the same essential organization of layers can be seen, with each layer of neurons characterized by particular axonal inputs and outputs and identifiable neuron types. For example, the outermost two cell layers of the cortex, layers 2 and 3, are composed principally of asymmetric neurons called pyramidal cells, whose axons make long-distance interarea and short-distance local recurrent connections within the cortex itself. The larger pyramidal cells of layer 5 get axonal input from the cortex but send projections outside the cortex to diverse regions such as the midbrain, the basal ganglia of the forebrain, and the spinal cord. Computational theories that focus on overall cortex uniformity have offered detailed conjectures on the “canonical computation” enacted by the cortex over its whole surface (e.g., [2,3]).

On the other hand, the cortex also presents a striking mosaic of variability: the entire surface is tiled into a number of “areas,” with each area containing sensory, motor, cognitive, or other computed dimensions laid out in an orderly topographic manner (such as the primary motor or visual cortex, common to all species) as well as the sensory and motor specializations particular to each, ranging from electroreception in the platypus to trunks in elephants [4]. Systematic regional differences related to sensory versus motor processing requirements [5] or neuroembryological gradients that progressively reorganize brains of different sizes in a predictable manner have also been proposed [6].

How a computational device scales also depends on what it is made up of. The cell (or in the case of the brain, neurons and glia) is the building block of the organism. In general, larger organisms (in this case, mammals) are made of more cells, not larger cells. In addition, fundamental cellular processes are largely scale invariant—in the case of the neuron, relevant examples of general scale invariance are the duration of the action potential and the fundamental biophysics of the propagation of action potentials down axons. The neuron represents a special problem for scaling at the cell level: while the cell bodies of neurons have a minimal relationship to the whole-organism mass in mammals, the axons may need to traverse distances varying directly with brain or body size [7]. For example, comparable single neurons relaying information about the body midline across the cortex must travel millimeters in mice, but centimeters in macaque monkeys; to relay a motor command from the cortex to the spinal cord requires millimeters of travel in mice, but meters in giraffes. Though the speed of axonal transmission can be substantially increased by axon myelination, and is a solution to increasing brain volume, myelination adds mass to the nervous system, eventually exacerbating the problem it attempts to solve. So, with the affordances and constraints of these building blocks, how has evolution scaled up the cortex over five orders of magnitude?

Kennedy, along with Toroczkai, Ercsey-Ravasz, and their colleagues [8,9], have provided an exceptionally interesting piece of information to both the puzzle of cortex scaling and cortex function. To this end, in a new study published in *PLOS Biology*, they have compared two relatively new comprehensive data sources from two species, the rhesus macaque and the laboratory mouse [8]. The first is a comprehensive analysis of the connectivity of the cortex of the rhesus macaque, as determined by neuroanatomical tracing studies (as contrasted with functional or structural connectivity determined by various imaging methods), done by the same group [10], and the second is a similar study done in mice [11,12]. Tracer injections made systematically across the cortical surface and in each cortical area determined the number of areas each cortical area projects to and receives projections from, with these tracer techniques exploiting the normal intracellular transport mechanisms of neurons. New to this study, the absolute axon length and cortical location each projection represents was added to the analysis. Over what range and with what pattern does a comparable location in cortex in mice versus monkeys project? Does the range reflect the surface area of the cortex, the growth constraints of neurons, or the idiosyncratic specializations of each species?

Before the answer, a few basic facts about what is known about the scaling of the cortex should be put in play (Fig 1). As the cortex increases in volume, it principally increases in surface area, producing the characteristic folding of a large cortex, but the depth of the cortex increases as well; in the case of the mouse versus monkey, the surface area of the mouse is very roughly 100 mm^2^ and the monkey 20,000 mm^2^, while the mean depth of the cortex in the mouse is about 1–2 mm and the monkey 2–3 mm. Cortical area and depth reflect variable contributions of neuron number and connection volumes, both related to the order of neurogenesis and developmental duration [13]. All mammals possess primary visual, auditory, and sensorimotor cortices, no matter how small the brain [4], and after that, the number of cortical areas scales regularly with overall cortical surface area [14]. Therefore, the greatest increase in number of cortical areas comes from the “association” regions of parietal, temporal, and frontal areas. Connections within the cortex are predominantly made to immediately adjacent regions—with a lesser fraction of long-distance connections—in any size of brain (reviewed in [8]). The proportion of local connections compared to long-distance connections increases in larger brains, which is thought to be related to intractable problems of accommodating larger and larger volumes of interconnecting axons.

**Fig 1:**
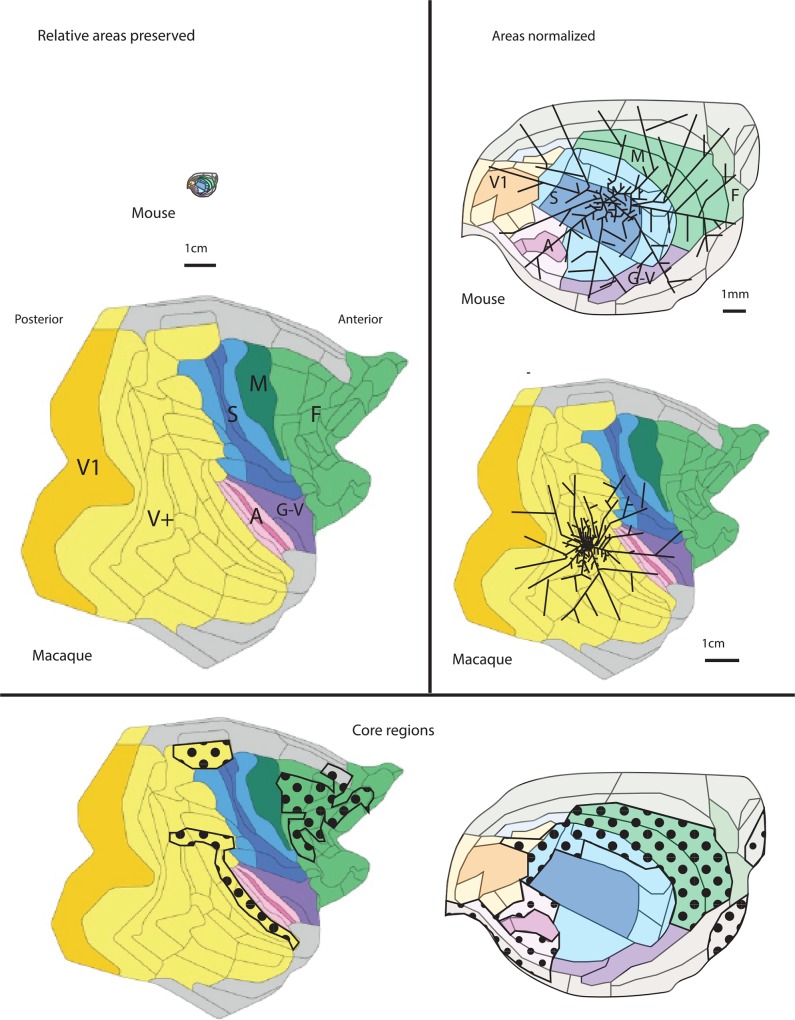
Scaling axon distribution in mouse and macaque isocortex. **Top left:** Lateral view of mouse (top) versus macaque monkey brain (bottom), drawn to the same scale to demonstrate relative size. Dimensions are approximate, redrawn from “unrolled” representations of brain schematics [6,8,9], and broad cortical regions are color-coded. Saturated areas represent primary and motor areas, and less-saturated regions represent secondary regions. Primary and secondary visual cortex are shown in yellow. S, somatosensory, blue; M, motor cortex, green; F, frontal or premotor cortex, light green; A, auditory, pink; G–V, gustatory-visceral or insular, purple; limbic, orbital, and olfactory, gray. Strict mouse-to-monkey area homologies should not be presumed. **Top right:** The mouse and monkey cortex are here normalized to present approximately the same lateral area. On each is superimposed a schematic of the axon distribution emanating from a single cortical point, drawn to demonstrate the properties described in [8,9]. These are: (1) equivalent absolute axon densities in the central region of the projection, (2) faster falloff in connection density in the distal regions of the monkey axonal projections, and (3) lesser relative coverage of the entire cortical surface from a single tracer injection site in the monkey. **Bottom**: Interesting equivalence of the “core regions” of the cortex (dotted) in normalized monkey and mouse cortices.

The researchers for this paper [8] evaluated the network properties of cortical areas of the monkey versus the mouse cortex using the mathematics of graph theory, describing graphs of mouse and monkey cortical connections with respect to three essential properties. First, the graphs were “directed,” as the direction of connectivity from one “node”—in this case a tracer injection in a cortical area—to another was explicit in the method. Second, they were “weighted,” taking into account the relative numbers of projections from each area to any other. Finally, the nodes of the graphs, identical with tracer injection sites, were “spatially embedded”—that is, assigned relative locations in the cortex defined as the absolute distances between areas of cortex that axons would have to traverse. Prior graphical analyses of network structure, assessed from multiple neuroanatomical and imaging methods, have evaluated directed and weighted but not spatially embedded data corpora. In both the monkey and mouse, the distribution of axonal connection lengths as a function of distance exhibits an exponential decay, independently of area definition. The exponential decay rate along with distance distributions was found to determine the probability of a connection of any two cortical areas. The central question of the study is to explore the extent that the features of cortical connectional organization are predicted by this exponential falloff rule.

To get a better feel for the physical proportions of the two cortices, and the connection problem to be solved, the surface area of the cortex of the rhesus macaque is roughly 200 times greater than a mouse’s (Fig 1, top left). The outside range of axon extent from a single injection is about 50 mm in the monkey and 10 mm in a mouse [8]. In the mouse, this range is adequate for any single cortical location to potentially extend to any other cortical location (with declining likelihood with distance), but the outside range for the monkey fails to reach the full cortical extent by about half (Fig 1, top right). This shortfall in coverage is exacerbated by the fact that the falloff of the probability of making longer connections falls off more steeply in the monkey compared to the mouse when the two brains are scaled to a common template (Fig 1, top right, this paper; Fig 7 a, b in [8]). Interestingly, the falloff in connection probability for short-range, likely unmyelinated local connections (about 1.5 mm) is identical in the two species.

Even with this fundamental physical disparity, many features of connectivity are similar in the two species, some of which are predictable by the spatially explicit exponential distance falloff rule of connectivity. Both exhibit “cliques” of cortical areas, completely connected sets of areas that can be used to define “core” and “peripheral” areas. In the monkey, such highly interconnected cliques and core regions are found principally in the frontal and parietal cortex and exclude primary sensory and motor areas; in the mouse, the overall spatial distribution of clique regions is similar but includes some primary sensory and motor cortical regions (Fig 1, bottom). The distribution of “motifs”—the pattern of forward, backward, and reciprocal connections between areas—is well predicted by the exponential distance rule and is similar in some interesting respects in the two species. Despite great evolutionary time and massive volume changes, it appears that the cortex has evolved to retain an integrative structure and not devolve into a collection of computationally-isolated special function devices.

Notable deviations from an all-inclusive, distance-based rule of connectivity appear in both species as well—“association” areas are more likely to have reciprocal interconnections than “canonical” primary sensory and motor areas, independent of how closely they neighbor each other. This feature is less pronounced in the mouse but still present. Much variation remains to be explained, particularly that which might be produced by evolutionarily specified or experientially-stabilized connections. For example, if coactivation of particular modalities serves to preferentially stabilize the long-distance connections serving them (Hebbian “fire-together, wire together”), as in the case of auditory-vocal-visual coactivation in animals capable of vocal learning, the probability of connections between them would be greater than that given by a fundamental distance rule scaffolding alone.

The authors point out that the low probability of long-distance connections may make those connections particularly vulnerable in large brains, citing evidence of this kind in both autism and Alzheimer’s disease. This example, while well taken, brings up the caution that not every problem caused by connectivity (or any particular process) should be expected to be solved by connectivity, as multiple alterations in axon caliber and myelination varying with brain size have been described as partial solutions to this problem [15,16].

Finally, how a computational device scales may take advantage of how it is made (or, be constrained by how it is made). The exponential distance rule points to a potential simple source of developmental scaffolding for cortical connections in the initial outgrowth of intracortical axons. Initial axon outgrowth, moreover, has some distinct features within and between species that should be investigated for their role in later network architecture. The frontal cortex is the first to send out axons as the cortex develops [6]: does this cause its position in “cliques” of cortical connectivity and its core status? Is the duration of development, though much longer in monkeys than mice, the effective limiting factor that determines how far intracortical axons have time to extend [17]? Evolution has likely filtered from the original range of developmental rules organizing the cortex those rules generating computational architectures robust to common challenges. Variation in the sizes of animals and all their parts is certainly one of those repeated challenges.

## References

[1] Finlay BL, Darlington RB. Linked regularities in the development and evolution of mammalian brains. Science. 1995; 268(5217): 1578–1584. 7777856 10.1126/science.7777856

[2] Miller KD. Canonical computations of cerebral cortex. Current Opinion in Neurobiology, 2016; 37: 75–84. 10.1016/j.conb.2016.01.008 26868041 PMC4944655

[3] Bastos AM, Usrey WM, Adams RA, Mangun GR, Fries P, Friston KJ. Canonical microcircuits for predictive coding. Neuron, 2012; 76(4): 695–711. 10.1016/j.neuron.2012.10.038 23177956 PMC3777738

[4] Krubitzer L. In search of a unifying theory of complex brain evolution. Annals NY Acad Sci. 2009: 1156: 44–67. 10.1111/j.1749-6632.2009.04421.x PMC2666944 19338502

[5] O'Reilly RC, Herd SA, Pauli WM. Computational models of cognitive control. Current Opinion in Neurobiology, 2010; 20(2), 257–261. 10.1016/j.conb.2010.01.008 20185294 PMC2862817

[6] Finlay BL, Uchiyama R. Developmental mechanisms channeling cortical evolution. Trends Neurosci. 2015;38(2):69–76. 10.1016/j.tins.2014.11.004 25497421

[7] Kaas J. Why is brain size so important: design problems and solutions as neocortex gets bigger or smaller. Brain and Mind. 2000;1:7–23.

[8] Sz Horvát, Gămănuț R, Ercsey-Ravasz M, Magrou L, Gămănuț B, Van Essen DC, et al Spatial Embedding and Wiring Cost Constrain the Functional Layout of the Cortical Network of Rodents and Primates. PLoS Biol 2016 14(7): e1002512 10.1371/journal.pbio.1002512 27441598 PMC4956175

[9] Ercsey-Ravasz M, Markov NT, Lamy C, Van Essen DC, Knoblauch K, Toroczkai Z, et al A predictive network model of cerebral cortical connectivity based on a distance rule. Neuron 2013 80, 184–197. 10.1016/j.neuron.2013.07.036 24094111 PMC3954498

[10] Markov NT, Ercsey-Ravasz MM, Ribeiro Gomes AR, Lamy C, Magrou L, Vezoli J, et al A weighted and directed interareal connectivity matrix for macaque cerebral cortex. Cereb Cortex. 2014;24(1):17–36. 10.1093/cercor/bhs270 23010748 PMC3862262

[11] Oh SW, Harris JA, Ng L, Winslow B, Cain N, Mihalas S, et al A mesoscale connectome of the mouse brain. Nature. 2014;508(7495):207–14. 10.1038/nature13186 24695228 PMC5102064

[12] Zingg B, Hintiryan H, Gou L, Song MY, Bay M, Bienkowski MS, et al Neural networks of the mouse neocortex. Cell 156, 1096–1111, doi: 10.1016/j.cell.2014.02.023 (2014) 24581503 10.1016/j.cell.2014.02.023 PMC4169118

[13] Cahalane DJ, Charvet CJ, Finlay BL. Modeling local and cross-species neuron number variations in the cerebral cortex as arising from a common mechanism. Proc Natl Acad Sci U S 1002 A. 2014;111(49):17642–7. 10.1073/pnas.1409271111 PMC4267349 25422426

[14] Finlay BL, Brodsky PB. Cortical evolution as the expression of a program for disproportionate growth and the proliferation of areas In: Evolution of Nervous Systems 2006 Kaas J.H., editor. Elsevier: Oxford University Press pp. 73–96

[15] Wang SS-H, Shultz JR, Burish MJ, Harrison KH, Hof PR, Towns LC, et al Functional trade-offs in white matter axonal scaling. 2008; J Neurosci, 28(15): 4047–4056. 10.1523/JNEUROSCI.5559-05.2008 18400904 PMC2779774

[16] Innocenti GM, Vercelli A, Caminiti R. The diameter of cortical axons depends both on the area of origin and target. Cerebral Cortex, 2014: 24(8), 2178–2188. 10.1093/cercor/bht070 23529006

[17] Workman A, Charvet CJ, Clancy B, Darlington RB, Finlay BL. Modeling transformations of neurodevelopmental sequences across mammalian species. J Neurosci 2013: 17: 7368–7383 10.1523/JNEUROSCI.5746-12.2013 PMC3928428 23616543

